# Obesity and Sex Affect the Immune Responses to Tick-Borne Encephalitis Booster Vaccination

**DOI:** 10.3389/fimmu.2020.00860

**Published:** 2020-05-27

**Authors:** Erika Garner-Spitzer, Eva-Maria Poellabauer, Angelika Wagner, Angela Guzek, Ines Zwazl, Claudia Seidl-Friedrich, Christoph J. Binder, Karin Stiasny, Michael Kundi, Ursula Wiedermann

**Affiliations:** ^1^Institute of Specific Prophylaxis and Tropical Medicine, Medical University of Vienna, Vienna, Austria; ^2^Department for Laboratory Medicine, Medical University Vienna, Vienna, Austria; ^3^Center of Virology, Medical University Vienna, Vienna, Austria; ^4^Center for Public Health, Medical University Vienna, Vienna, Austria

**Keywords:** obesity, immune dysfunction, vaccination, tick-borne encephalitis, sex, metabolism, hormones

## Abstract

Obesity has dramatically increased over the last 30 years and reaches according to World Health Organization dimensions of a global epidemic. The obesity-associated chronic low-level inflammation contributes to severe comorbidities and directly affects many immune cells leading to immune dysfunction and increased susceptibility to infections. Thus, prophylaxis against vaccine-preventable diseases is crucial, yet the responsiveness to several vaccines is unclear under obesity. In order to assess the responsiveness to tick-borne encephalitis (TBE) vaccine, we revaccinated 37 obese individuals and 36 normal-weight controls with a licensed TBE vaccine. Metabolic, hormonal, and immunologic profiles along with vaccine-specific humoral and cellular immune responses were evaluated in sera and peripheral blood mononuclear cells (PBMCs) before, 1 week, 4 weeks, and 6 months after TBE booster. Obese adults had significantly increased metabolic (triglycerides, cholesterol ratios, leptin, insulin) and proinflammatory (C-reactive protein) parameters. They showed stronger initial increase of TBE-specific Ab titers (d7_d28) followed by a significantly faster decline after 6 months, which correlated with high body mass index and leptin and insulin levels. The fold increase of Ab-titer levels was significantly higher in obese compared to control males and linked to reduced testosterone levels. Obesity also affected cellular responses: PBMCs of the obese vaccinees had elevated interleukin 2 and interferon γ levels upon antigen stimulation, indicating a leptin-dependent proinflammatory T_H_1 polarization. The expansion of total and naive B cells in obese might explain the initial increase of Ab titers, whereas the reduced B-memory cell and plasma blast generation could be related to fast Ab decline with a limited maintenance of titers. Among T follicular helper cell (Tfh) cells, the Tfh17 subset was significantly expanded particularly in obese males, where we observed a strong initial Ab increase. Systemic but not local vaccine side effects were more frequent in obese subjects as a possible consequence of their low-grade proinflammatory state. In summary, TBE booster vaccination was effective in obese individuals, yet the faster Ab decline could result in a reduced long-term protection. The sex-based differences in vaccine responses indicate a complex interplay of the endocrine, metabolic, and immune system during obesity. Further studies on the long-term protection after vaccination are ongoing, and also evaluation of primary vaccination against TBE in obese individuals is planned.

**Clinical Trial Registration:** NCT04017052; https://clinicaltrials.gov/ct2/show/NCT04017052.

## Introduction

The worldwide prevalence of obesity has tripled within the last 30 years, and in 2016, 1.9 billion people older than 18 years were overweight, with one-third of them classified as obese. This equals 13% of the world population, 11.1% of men and 15% of women. For adults, the World Health Organization (WHO) defines overweight as body mass index (BMI) ≥25 kg/m^2^ and obesity as BMI ≥30 kg/m^2^ (1). In 2016, 23.3% of Europeans were obese (2), and the prevalence of obesity among US adults was even more striking with 39.8% (3). Also, younger individuals worldwide are greatly afflicted because the rate of overweight and obesity among children and adolescents aged 5 to 19 years increased dramatically from 4% in 1975 to 18% in 2016, and 41 million children younger than 5 years were overweight or obese. The WHO has adopted policies to halt the global epidemic and aims to limit obesity by 2025 to the rates of 2010 (1).

The immune system and metabolic system have coevolved, and their mutual influence regulates the sharing of resources between metabolic energy conservation and required implementation of energy-consuming immune defense mechanisms (4). Adipose tissue as part of the metabolic system is the site of energy storage, that is, accumulation of lipids in adipocytes, and local innate and adaptive immune cells in cooperation with adipocytes and endothelial cells maintain metabolic homeostasis. The adipocytes in obese white adipose tissue (WAT) become hypertrophic due to increased fat storage leading to hypoxia and cell death. Proinflammatory signals from dead adipocytes attract M1 macrophages, and tumor necrosis factor α (TNF-α) and interferon γ (IFN-γ) secreted from proinflammatory invariant natural killer T cells and CD8^+^ T cells, respectively, induce further macrophage accumulation and activation. This, together with T_H_1-polarized CD4^+^ T cells, results in local type 1 inflammation and consequently low-grade systemic inflammation (5). Adipocytes secrete leptin, a hormone that regulates body weight via leptin receptors (LepRs) in the central nervous system, where it triggers decreased food intake. Leptin receptor is expressed on many immune cells, and thus, leptin directly influences the immune system. Leptin activates granulocytes, macrophages (M1 phenotype), dendritic cells, and natural killer (NK) cells and leads to increased naive T- and B-cell proliferation, decreased T regulatory cell (Treg) proliferation and T memory expansion, as well as T_H_1 and T_H_17 polarization (6). In obese individuals, leptin levels are permanently high, because leptin signaling is impaired due to leptin resistance (7).

Obesity has severe health consequences such as insulin resistance and type 2 diabetes, hypertension, and dyslipidemia leading to atherosclerosis and cardiovascular disease (8), and an increased risk of certain types of cancer (9). Importantly, obesity also has a substantial impact on immunity and pathogen defense. Because of fat accumulation in bone marrow and thymus, the generation and output of naive immune cells are affected, leading to altered leukocyte populations and expansion of T cells and macrophages with predominantly inflammatory phenotype. These alterations impair immune defense, and consequently, obese individuals show increased susceptibility to infections (10). It has been shown that obese individuals are at greater risk of particularly respiratory tract infections caused by bacteria (11) and viruses (12). Furthermore, obesity was shown to pose a risk factor for increased morbidity and mortality after infection with the pandemic influenza A (H1N1) virus in 2009 (13), and impaired antiviral responses in the obese host are described in detail (14).

Given the increased susceptibility to infections in obese individuals, vaccinations as preventive measures are of crucial importance, but sufficient vaccine responses in obese individuals are questionable. Obesity is described as a clear risk factor for non-responsiveness to hepatitis B vaccination (15–18), and also reduced vaccine responses to hepatitis A virus vaccine were reported (19, 20). Obese adolescents mounted reduced antitetanus antibodies (Abs) (21), and overweight/obese adults had insufficient Ab titers to rabies vaccine 2 years postvaccination (22). In contrast, a more recent study showed that obese subjects mounted higher neutralizing antirabies Ab titers 4 weeks after vaccination (23). With respect to influenza, it is known that obesity is an important risk factor for severe disease complications, and therefore short- and long-term protection after influenza vaccination has been investigated: increased titers to trivalent influenza vaccine were observed in obese children (24), and also adults showed a higher initial fold increase of Ab levels after 4 weeks. However, this was followed by a faster decline after 12 months, and both were correlated with high BMI. Additionally, obese vaccinees showed a defective activation of CD8^+^ T cells with reduced granzyme B and perforin production (25).

Based on reports that long-term protection to influenza appears limited under obese conditions, we further asked whether these immunologic effects also apply to other vaccines such as tick-borne encephalitis (TBE). Tick-borne encephalitis virus is highly endemic in Austria, and ~90% of the population is vaccinated against TBE (26), yet no data are available on the effectiveness of TBE vaccine (adjuvanted, inactivated whole virus) in the continuously growing population of severely obese individuals. According to Austrian guidelines, TBE booster is required 5 years after primary vaccination (aged <60 years), and thus we wanted to investigate (a) if adequate booster responsiveness is present in a severely obese cohort, (b) if vaccination has an impact on the metabolic state, and (c) if vaccine reactogenicity is altered in the context of obese low-grade inflammation. We enrolled obese adults and normal-weight controls of both sexes and evaluated their metabolic/inflammatory parameters and sex hormones; moreover, vaccine-specific Ab titers and cellular responses were determined before and after TBE booster administration. Our goal was to determine whether obesity negatively influences TBE vaccine responsiveness and if there is a requirement for specific vaccination schedules in this risk population.

## Materials and Methods

### Study Design and Study Subjects

We investigated two parallel groups in an open-label, non-randomized, phase IV clinical trial with 37 obese (BMI >30 kg/m^2^) and 36 normal-weight adults (BMI <25 kg/m^2^). The study subjects were between 18 and 60 years old, and the groups were matched with respect to age and sex. All included participants had received a documented primary TBE vaccination and at least one booster. After providing written informed consent, a TBE booster vaccination was administered to the study participants. Venous blood was drawn prior to and 1 week, 4 weeks, and 6 months after vaccination, and the obtained sera were evaluated for TBE-specific antibodies. Furthermore, metabolic, hormonal, and immunologic parameters were determined in sera taken before and 4 weeks after vaccination. Sera were obtained from the antecubital vein between 8:00 and 10:00 am after an overnight fast. Peripheral blood mononuclear cells (PBMCs) were prepared prior to and 1 week after booster to assess the cellular immune responses. Furthermore, the study subjects reported occurrence, duration, and intensity of any local and systemic reactions in a diary during the week after vaccination. The study was approved by the ethical committee of the Medical University of Vienna (ECS Nr. 1179/2014) and the national regulatory authorities and registered at ClinicalTrials.gov (NCT04017052).

### Vaccines

The study subjects received the TBE vaccine FSME-IMMUN® 0.5 mL containing 2.4 μg inactivated TBE virus (strain Neudoerfl) and 0.35 mg Al(OH)_3_ by intramuscular injection into the muscle deltoideus with regular-length needles. The used lots VNR1N16E, VNR1Q10A, and VNR1P10E of FSME-IMMUN® vaccine were equally divided between obese and control groups. FSME-IMMUN® 0.5 mL (Pfizer Corporation Austria GmbH, Vienna, Austria) is licensed in Austria since 1996. All vaccines were stored at 2 to 8°C until usage.

### Preparation and Storage of Serum and PBMCs

Peripheral blood mononuclear cells were prepared from heparinized blood by Ficoll Paque centrifugation, as previously reported (27) and resuspended in RPMI 1640 containing 50% FCS (both Biochrom, Berlin, Germany) and 10% dimethyl sulfoxide (Merck, Darmstadt, Germany) for storage in liquid nitrogen until evaluation. Serum was obtained from native venous blood and stored at −20°C until evaluation, and an additional sample was sent to an external clinical laboratory for analyses of clinical chemical parameters and hormones (*labors.at*, Vienna, Austria).

### Metabolic and Inflammatory Parameters

Concentrations of total cholesterol, triglycerides, high-density lipoprotein (HDL) cholesterol, apolipoproteins A1 and B, lipoprotein A, glucose, fructosamine, leptin, insulin, and high-sensitivity C-reactive protein (CRP) were evaluated prior to vaccination and the measurements of cholesterol, triglycerides, HDL cholesterol, glucose, fructosamine, insulin und high-sensitivity CRP (hsCRP) were repeated 4 weeks thereafter. Low-density lipoprotein cholesterol was calculated according to the Friedewald formula, and the cholesterol ratio was calculated as total cholesterol/HDL cholesterol.

All analyses were done at the clinical laboratory *labors.at* in Vienna, Austria. Measurement of total cholesterol, triglycerides, HDL cholesterol, apolipoprotein A1 and B, glucose, and hsCRP was performed on Cobas C701 (Roche Diagnostics, Mannheim, Germany) according to the manufacturer's instructions. Fructosamine and lipoprotein A were measured on Cobas C501 and insulin on Cobas E602 (both Roche Diagnostics) according to the manufacturer's instructions. Leptin was quantified by RIA (Leptin RIA LEP-R44; Mediagnost, Reutlingen, Germany) according to the manufacturer's instructions.

### Hormones

The following sexual hormone levels were tested in serum prior to booster vaccination: testosterone, estrogen, progesterone, follicle-stimulating hormone (FSH), and luteinizing hormone (LH). Analyses were performed at the clinical laboratory *labors.at* in Vienna, Austria, using Cobas E602 (Roche Diagnostics) according to the manufacturer's instructions.

### TBE-Specific Neutralization Test Titers

Tick-borne encephalitis–specific neutralizing antibody titers were evaluated in serum by virus neutralization test (NT), performed according to Adner et al. (28) with TBE virus strain Neudoerfl at Pfizer Corporation Austria GmbH; Pfizer laboratory received anonymized serum samples for NT testing. The geometric mean titers (GMTs) were assessed before, 1 week, 4 weeks, and 6 months after vaccination.

### TBE-Specific *in vitro* Restimulation of PBMCs

Peripheral blood mononuclear cell samples stored in liquid nitrogen were reestablished in culture medium RPMI 1640 supplemented with 10% human AB serum (Biochrom) and 2 mM l-glutamine, 50 μM 2-mercaptoethanol, and 0.1 mg/mL gentamycin (all Sigma Aldrich, St. Louis, MO, USA). Cells were plated in 96-well round-bottom plates at 8 × 10^5^/well in duplicates and cultured with *TBE TICOVAC-like* antigen (0.096 μg/well), superantigen *Staphylococcus* enterotoxin B (SEB, 0.2 μg/well), and in culture medium only to assess cytokine baselines (200 μL total culture volume). Cultures were maintained for 48 h (37°C, 5% CO_2_, 95% humidity), and thereafter supernatants were harvested, pooled, and stored at −20°C until analyses.

### Quantification of Cytokine Production in Culture Supernatants

Cytokines interleukin 2 (IL-2), IFN-γ, IL-10, IL1-β, IL-6, IL-17, and TNF-α were quantified in culture supernatants from restimulated PBMCs obtained before (d0) and 7 days after vaccination (d7) using a Luminex 200 platform and Human Cytokine A Premix-Kit (Bio-Techne Ltd., Abingdon, UK) as previously described (27). All cytokine data are TBE-or SEB-specific concentrations minus the respective baseline levels measured in media-stimulated cultures.

### Flow Cytometric Lymphocyte Analyses

Peripheral blood mononuclear cells were surface stained with the fluorochrome-conjugated monoclonal antibodies listed below and stained intracellularly with monoclonal antibodies (mAbs) against transcription factor FOXP3 for characterization of Tregs. Data were acquired on a FACS Canto II flow cytometer by gating on cells with forward/side light scatter properties of lymphocytes and analyzed with FACS Diva 8.0 software (BD Biosciences, San Jose, CA, USA).

For PBMCs' surface staining, the following monoclonal antibodies were used: anti–human CD3 PE-Cy5 (clone HIT3a), anti–human CD4 APC-H7 (clone L200), anti–human CD8 APC (clone RPA-T8), anti–human CD28 BV510 (clone CD28.2), anti–human CD45RA BV421 (clone HI100), anti–human CD19 FITC (clone HIB19), anti–human CD27 PE (clone L128), anti–human CD38 PerCP-Cy5.5 (clone HIT2), anti–human CD24 BV421 (clone ML5), anti–human CD10 BV510 (clone HI10a), anti–human immunoglobulin D (IgD) PE-Cy7 (clone IA6-2), and anti–human IgM APC (clone G20-127), all from BD Biosciences; anti–human chemokine receptor 7 (CCR7) FITC (clone 150503) was obtained from R&D Systems, Inc. (Minneapolis, MN, USA). Regulatory T cells were characterized with anti–human CD3 PE-Cy5 (clone HIT3a), anti–human CD4 FITC (clone RPA-T4), anti–human CD25 PE (clone M-A251), anti–human CD45RA BV510 (clone HI100) (all BD Biosciences), and intracellular anti–human FOXP3-APC (clone PCH101; eBioscience, now Thermo Fisher Scientific, Waltham, MA, USA). Foxp3/Transcription Factor Staining Buffer Set (eBioscience) was used for the fixation/permeation procedure, and specific staining was verified with respective isotype controls. Natural killer T cells were characterized as CD3^+^/CD4^−^/CD8^−^ and were calculated as % difference of CD4 plus CD8 T cells to total CD3^+^ T cells.

T follicular helper cells (Tfh) were characterized by use of anti–human ICOS FITC (clone ISA3) and anti–human CXCR5 PE Cy7 (clone MU5UBEE) from eBioscience, now Thermo Fisher Scientific, as well as with anti–human PD-1 PE (clone EH12.2H7) and anti–human CXCR3 APC (clone G025H7) from BioLegend, San Diego, CA, USA. Furthermore, anti–human CD3 PerCP Cy5.5 (clone Ucht1), anti–human CD4 APC (clone L200), anti–human CCR6 BV421 (clone 11A9), anti–human CD45RA BV510 (clone HI100), and anti–human CD56 BV510 (clone NCAM16.2), all from BD Biosciences, were used.

### Total IgA

Serum concentrations of total IgA were determined before and 4 weeks after TBE booster vaccination. An *in vitro* diagnostic assay for the quantitative determination of IgA in human serum by means of immune nephelometry on the Atellica® NEPH 630 System was used. Measurements were carried out at the Department for Laboratory Medicine, Medical University of Vienna.

### Statistical Evaluation

Sample size determination was based on the assumption that a relative TBE titer difference of 1:2 should be detected at the two-sided significance level of 5% with a power of 80% given the standard deviation of log titers from Loew-Baselli et al. (29). To reach this goal, sample size for each group must be greater than or equal to *n* = 37. The primary endpoint (log TBE titer) was statistically evaluated by analysis of variance (ANOVA) with linear contrasts for the comparison of fold increases/decreases. Serum concentrations of cytokines as well as hormones and lipids were log transformed and fractions of PBMCs were square root transformed before analysis and evaluated exploratory like the primary endpoint by ANOVA and linear contrasts. In all analyses, residuals were tested for normality by Kolmogorov–Smirnov tests with Lilliefors' corrected *p*-values. Homogeneity of variances was tested by Levene tests. Blood parameters are shown in graphs with geometric means and 95% confidence intervals (CIs). Cell fractions are shown as dot plots with arithmetic means. Relationship between blood parameters and TBE titers (and fold increase/decrease) was investigated by Spearman rank correlations. All analyses were performed using SPSS 25.0 (IBM Corp., Armonk, NY, USA), and graphics were prepared by GraphPad Prism (version 7.00; GraphPad Software, San Diego, CA, USA).

## Results

### Cohort

The investigated groups were obese subjects (*n* = 37) and normal-weight controls (*n* = 36) (Supplementary Figure 1). The demographic parameters age and sex were similar between the groups, as was the interval since the last TBE booster (~8 years) and the number of previously received booster vaccinations. The average BMI in the obese group was 38.9 vs. 22.1 kg/m^2^ in healthy controls (*p* < 0.0001), and also weight (119.1 vs. 68.1kg) and waist-to-hip ratio (0.94 vs. 0.83, both *p* < 0.0001) were significantly different between the groups (Table 1). All subjects fulfilled the general study eligibility criteria (Supplementary Table 1); individuals diagnosed with type 1 diabetes mellitus (classified as an autoimmune disease) were not eligible for the study, and five subjects in the obese group suffered from type 2 diabetes. Of female participants in both groups, ~50% had a normal menstrual cycle, ~25% used hormonal contraceptives, and ~25% were postmenopausal.

**Table 1:** Demographic data of study subjects.

	**Obese**	**Controls**
Participants (*n*)	37	36
Sex (m/f)	17/20	16/20
Mean age (y)	46.0 (43.2–48.8)	45.4 (42.0–48.8)
Interval to last booster (y)	8.5 (6.7–10.4)	8.0 (5.9–10.1)
Previous TBE boosters (*n*)	2.6 (1.8–3.4)	2.5 (1.8–3.2)
Weight (kg)^****^	119.1 (112.6–125.5)	68.1 (64.9–71.2)
BMI^****^	38.9 (37.2–40.6)	22.1 (21.4–22.8)
Waist-to-hip ratio^****^	0.94 (0.91–0.98)	0.83 (0.81–0.86)

*Values are given as arithmetic means with 95% CI. Abbreviations: y, year; f, female; m, male; kg, kilogram; BMI, body mass index. Analysis of variance with linear contrasts*.

**** *p ≤ 0.0001*.

### Metabolic and Inflammatory Parameters and Hormones

**Figure 1:**
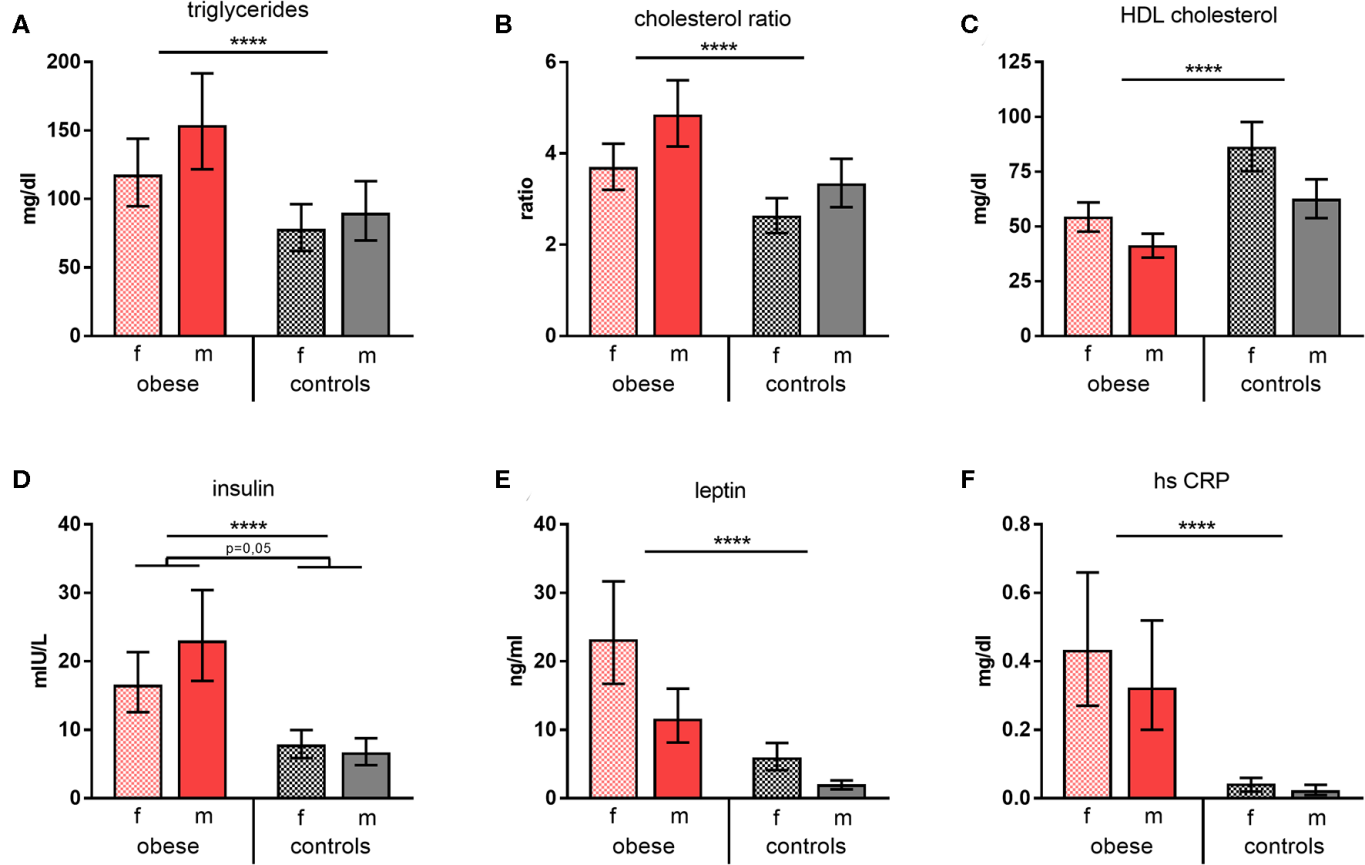
Metabolic and inflammatory parameters. Concentrations/values of **(A)** triglycerides (mg/dl), **(B)** cholesterol ratios, **(C)** HDL cholesterol (mg/dl), **(D)** insulin (mIU/L), **(E)** leptin (ng/ml), and **(F)** high-sensitive CRP (mg/dl) as geometric means (GM) with 95% CI; cholesterol ratio was calculated as total cholesterol/HDL cholesterol; values for females (f) and males (m). ANOVA with linear contrasts; ****p ≤ 0.0001.

Triglycerides and cholesterol ratios (total cholesterol/HDL cholesterol) were significantly increased in obese subjects, whereas HDL cholesterol was significantly decreased (Figures 1A–C). Furthermore, leptin and also insulin were present in significantly higher concentrations in obese individuals vs. normal-weight controls; of note, obese males featured disproportionally high insulin levels. Measurement of glycemic parameters showed higher glucose and decreased fructosamine levels in the obese cohort (Supplementary Table 2). High-sensitivity CRP was determined to assess systemic inflammation and was significantly increased in the obese group (Figures 1D–F). No substantial changes of the metabolic parameters tested 4 weeks after booster were observed. Females of both groups presented with a slight reduction of some of the respective parameters, whereas this was not the case for males (Supplementary Table 2).

**Figure 2:**
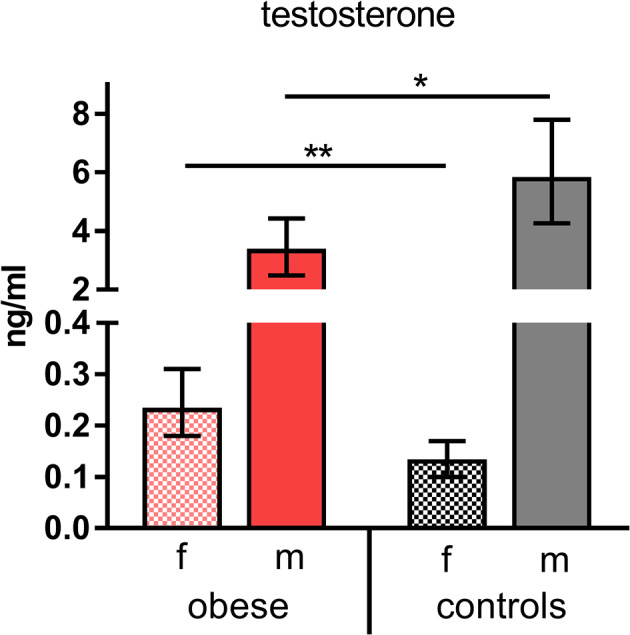
Testosterone concentrations. Testosterone (ng/mL) indicated as geometric mean (GM) concentrations with 95% CI; values for females (f) and males (m). Analysis of variance with linear contrasts; **p ≤ 0.01, *p ≤ 0.05.

Taking into consideration that WAT not only produces adipokines, but is as well involved in metabolic processing of sex hormones (i.e., testosterone), we measured concentrations of the most important hormones in our study population. Female hormone levels of estradiol, progesterone, LH, and FSH showed the expected physiologic differences between sexes without significant changes in obese vs. control subjects (Supplementary Table 3). In contrast, testosterone levels were significantly reduced in obese males and increased in obese females compared to the respective normal-weight male and female controls (Figure 2).

### TBE-Specific NT Titers

**Figure 3:**
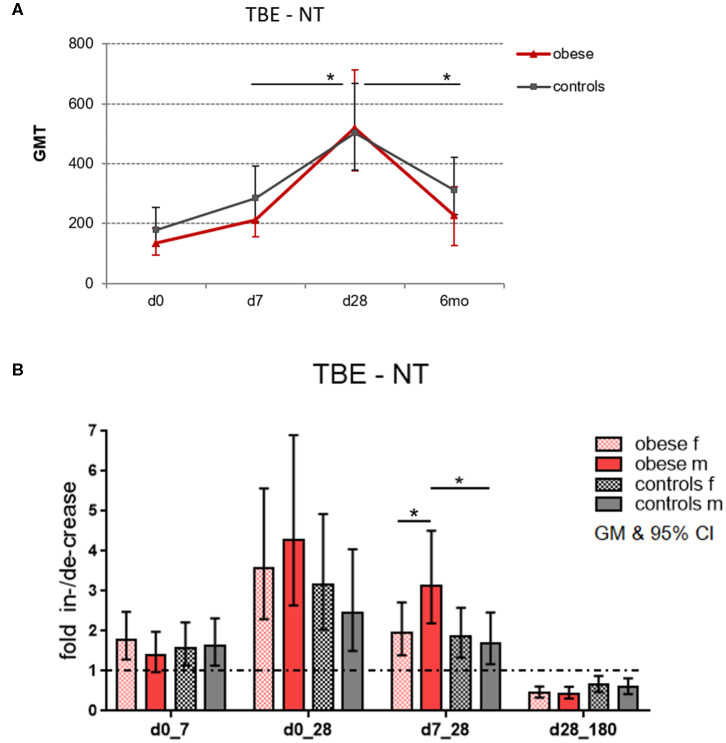
Geometric mean titers (GMT) of neutralization tests (NT). Anti-TBE virus antibody titers were measured by NT in sera obtained before (day 0) and 1 week (d7), 4 weeks (d28), and 6 months (d180) after booster. **(A)** Titer kinetics of neutralizing TBE-specific antibodies, GMT with 95% CI. The *p* values in the graph indicate the differences in increase and decline rates between obese and control groups. **(B)** Fold increase of GMT from d0 to d7, d0 to d28, and d7 to d28 and fold decrease from d28 to d180 (6 months) with 95% CI non-overlapping with 1, indicating a significant increase from baseline with *p* ≤ 0.05; values for males and females in obese and control cohort. Analysis of variance with linear contrasts; *p ≤ 0.05.

In order to assess the magnitude of vaccine-specific immune responses, neutralizing TBE-specific antibody titers (NT) were measured as a correlate of protection, and titers at dilutions >1:10 were considered protective/clinically meaningful (30). Healthy controls and obese subjects had protective GMTs before booster, but titers were higher in controls (GMT 178) than in obese subjects (GMT 134); the difference was, however, not statistically significant. One week after booster, Ab titers significantly increased by 1.6-fold in both groups. Highest titer levels were reached after 4 weeks with GMT 503 in the control and GMT 518 in the obese group. The obese group showed higher fold increase between d7 and d28 (*p* = 0.05) than controls, and also the subsequent decline at 6 months (to GMT 310 in controls and GMT 229 in obese) was significantly stronger in the obese cohort (*p* = 0.04; Figure 3A). When titers were analyzed according to sex, males in the obese group showed a stronger fold increase than females between d7 and d28 (*p* = 0.05), and in accordance, titer increases from d7 to d28 were significantly higher in obese vs. control males (Figure 3B). No significant sex differences in fold increases were observed in controls.

### Correlation of NT Titer Levels With Metabolic Parameters and Hormones

Initial NT titer levels (d0) and titer increases from d0 to d28, from d7 to d28, and decrease from d28 to 6 months after booster were correlated with some metabolic parameters and/or BMI for all study subjects. Spearman correlation showed that the respective fold increases of NT titers were positively correlated with BMI and metabolic parameters, whereas prebooster titers and decline rates were negatively correlated (Table 2A). When hormone levels in male and female subjects were related to the respective titer increases/decreases, testosterone concentrations in males showed a trend toward negative correlation with titer increases from d0 to d28, and positive correlation with baseline NT titers and decline rates. Similarly, LH in males showed negative correlation with titer increase and positive correlation with decline (Table 2B). In female subjects, the titer increases from d7 to d28 were positively correlated with LH and negatively with progesterone levels (Table 2C).

**Table 2:** Correlation of TBE NT titer kinetics with BMI, metabolic parameters, and hormones.

	**log NT at d0**		**NT increase d0_28**		**NT increase d7_28**		**NT decrease d28_180**	
	***r*_**s**_**	***p***	***r*_**s**_**	***p***	***r*_**s**_**	***p***	***r*_**s**_**	***p***
**(A) Correlation of log TBE NT titers at d0, log TBE NT titer increase d0_d28, log TBE NT titer increase d7_d28, and log TBE NT titer decline d28_d180 with body mass index (BMI) and metabolic parameters (insulin, leptin, triglycerides, cholesterol ratio, HDL cholesterol) measured before vaccination (d0)**.								
BMI	−0.20	0.085	0.28	0.017^*^	0.28	0.014^*^	−0.27	0.019^*^
Insulin	−0.24	0.038^*^	0.29	0.011^*^	0.29	0.012^*^	−0.26	0.024^*^
Leptin	−0.09	0.470	0.27	0.022^*^	0.24	0.041^*^	−0.18	0.115
TG	−0.23	0.052	0.20	0.086	0.11	0.341	−0.24	0.035^*^
Chol R	−0.23	0.050	0.09	0.408	0.05	0.638	−0.20	0.079
HDL	0.20	0.082	−0.10	0.391	0.00	0.997	0.23	0.050
**(B, C) Correlation of log TBE NT titers at d0, log TBE NT titer increase d0_d28, log TBE NT titer increase d7_d28, and log TBE NT titer decline d28_d180 with levels of testosterone, estrogen, progesterone, FSH, and LH measured before vaccination (d0) in (B) men and (C) women**.								
**(B) MALES**								
Testosterone	0.388	0.025^*^	−0.323	0.067	−0.203		0.339	0.053
Estrogen	−0.028		−0.066		−0.031		0.115	
Progesterone	−0.094		−0.025		0.194		−0.071	
FSH	0.057		−0.074		−0.016		0.114	
LH	0.091		−0.391	0.024^*^	−0.199		0.380	0.029^*^
**(C) FEMALES**								
Testosterone	−0.257		0.143		−0.125		−0.147	
Estrogen	0.124		−0.194		−0.125		−0.021	
Progesterone	−0.255		0.117		−0.315	0.048^*^	−0.093	
FSH	−0.036		0.266		0.286	0.073	−0.026	
LH	−0.116		0.242		0.337	0.033^*^	−0.055	

*Shown is Spearman correlation coefficient r_s_ (+ or −) and the respective p-value*;

* *p ≤ 0.05, r_s_ indicated in red*.

### Quantification of Cytokine Production in Restimulated PBMC Supernatants

**Figure 4:**
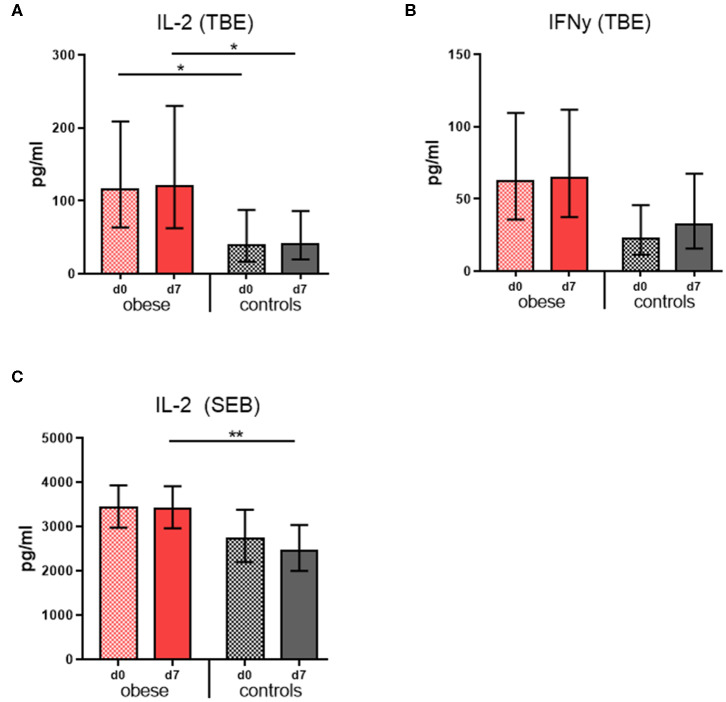
Cytokine production of re-stimulated PBMCs. Interleukin 2 and IFN-γ concentrations were measured in PBMC culture supernatants with Luminex technology. Geometric mean (GM) concentrations (pg/mL) with 95% CI from PBMCs obtained before (d0) and 1 week (d7) after booster are shown; cytokine concentrations obtained in media-only cultures were subtracted. **(A,B)** Interleukin 2 and IFN-γ concentrations (pg/ml) from PBMC incubated with 0.48 μg/mL TBE antigen for 48 h, and **(C)** IL-2 concentrations (pg/mL) from PBMCs incubated with 1 μg/mL *Staphylococcus* Enterotoxin B for 48 h. ANOVA with linear contrasts; **p ≤ 0.01, *p ≤ 0.05.

Cytokine concentrations were measured in culture supernatants of restimulated PBMCs in order to assess the cellular responses to TBE vaccine antigen. Tick-borne encephalitis–specific IL-2 concentrations were significantly increased in the obese group before booster compared to controls and remained so 1 week after booster with only marginal changes; also, mitogenic stimulation with SEB resulted in higher IL-2 levels in the obese group (Figures 4A,C). Similarly, TBE-specific IFN-γ concentrations were higher in obese vs. controls before booster (*p* = 0.06), and upon vaccination, a tendency toward higher IFN-γ levels was observed only in controls (**Figure 6B**). Quantification of proinflammatory cytokines IL1-β, IL-6, IL-17, and TNF-α upon mitogenic SEB stimulation showed a trend toward increased IL-17 production (*p* = 0.23) in the obese group before booster (Supplementary Figure 3B).

### Flow Cytometric Lymphocyte Analyses

In order to investigate whether the metabolic state of obesity influenced the distributions of lymphocyte subsets, we performed flow-cytometric analysis of PBMCs.

**Figure 5:**
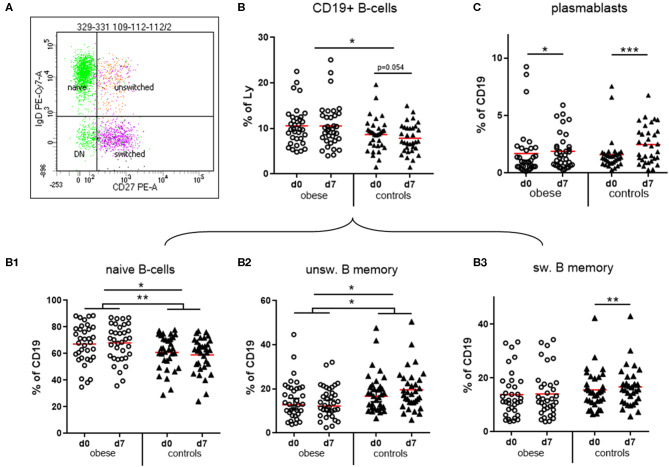
Quantification of naïve and memory sub-populations of B lymphocytes and plasma blasts. Peripheral blood mononuclear cells were stained with fluorochrome-labeled mAbs and analyzed on a BD FACS Canto II flow cytometer. **(A)** Scatterplot of CD19^+^ B cells with expression of IgD and CD27 to distinguish naïve, un-switched, switched and double negative (DN) subsets. **(B)** Quantification of total CD19^+^ B-cells and of **(B1)** naïve, **(B2)** un-switched, and **(B3)** switched subset before (d0) and one week (d7) after TBE booster vaccination; line is arithmetic mean. **(C)** Plasma blasts (CD19^+^/CD27^++^/CD38^high^) as percentages of total CD19^+^ B cells before (d0) and 1 week (d7) after booster; line is arithmetic mean. ANOVA with linear contrasts; ***p ≤ 0.001, **p ≤ 0.01, *p ≤ 0.05.

We observed that CD19^+^ B cells and CD3^+^ T cells as percentages of total lymphocytes were within normal ranges. The numbers of B cells were significantly increased in the obese group (Figure 5B), whereas T cells were rather decreased (Supplementary Figure 3A; *p* = 0.07).

#### B Cells

Four distinct subsets of B cells were distinguished by expression of CD27 and IgD: naive B cells are IgD^+^/CD27^−^, whereas CD27^+^ memory B cells can be either IgD^+^ (unswitched B-memory) or IgD^−^ after class switch to G, A, or E isotype (switched B-memory cells). The small double-negative population consists partly of class-switched, resting memory B cells lacking CD27 expression (31) (Figure 5A). Obese subjects had significantly more naive B cells and in turn significantly decreased IgD^+^ unswitched memory B cells compared to controls. Booster vaccination in controls was associated with a significant decrease of naive B cells and in turn increase of unswitched and switched (*p* < 0.01) B-memory cells after 1 week, whereas no progression to a B memory phenotype was observed in the obese group (Figures 5B1–B3). We observed a less pronounced expansion of plasma blasts (CD19^+^/CD27^++^/CD38^high^) in the obese group compared to normal-weight controls after vaccination (Figure 5C). No sex-specific differences regarding B-cell subsets and plasma blasts before and after booster were observed (Supplementary Table 4).

#### T Cells

**Figure 6:**
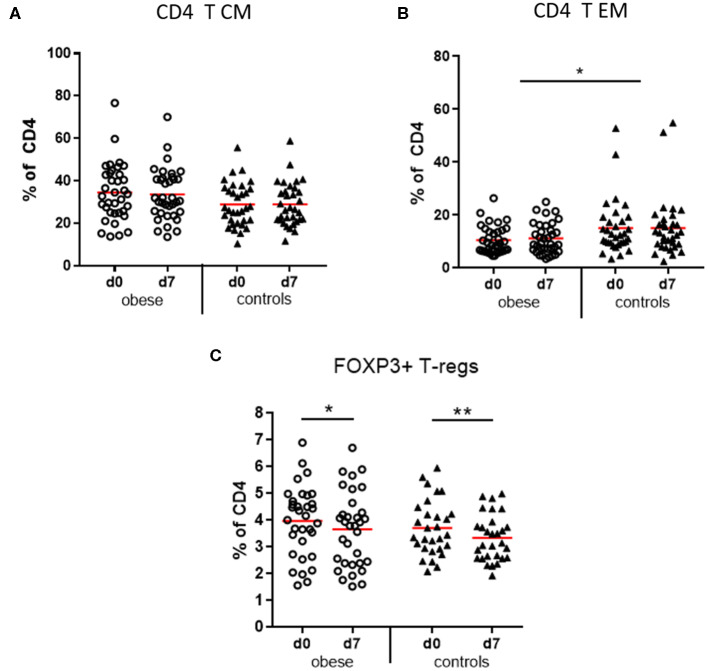
Memory subsets of CD4 T lymphocytes and T regulatory cells. Peripheral blood mononuclear cells were stained with fluorochrome-labeled mAbs and analyzed on a BD FACS Canto II flow cytometer. **(A)** Quantification of T central memory (T_CM_) and **(B)** T effector memory (T_EM_) subset before (d0) and 1 week (d7) after booster vaccination as % of total CD3^+^/CD4^+^ T cells; **(C)** T regulatory cells (Tregs, CD3^+^/CD4^+^/CD25^+^/FOXP3^+^) as percentage of total CD3^+^/CD4^+^ T cells before (d0) and 1 week (d7) after booster; line is arithmetic mean. Analysis of variance with linear contrasts; **p ≤ 0.01, *p ≤ 0.05.

CD3^+^ T cells as percentages of total lymphocytes tended to decrease in obese subjects vs. controls due to significant reduction of NK-T cells (*p* < 0.05) and a non-significant decrease of CD8^+^ T cells (*p* = 0.12). No difference was observed with respect to CD4^+^ T-helper cells (Supplementary Figures 2A–D). Four functionally different subsets of CD4^+^ and CD8^+^ T cells were quantified by expression of CD45RA and CCR7, which mediates migration to lymph nodes: naive T cells (T_N_), lymph node homing central memory T cells (T_CM_), peripheral tissue homing effector memory T cells (T_EM_), and a CD45RA re-expressing effector memory subset (T_EMRA_) (32, 33). The obese group showed a trend to more CD4 T_CM_ cells (*p* = 0.067) vs. significantly reduced T_EM_ cells (*p* < 0.05) compared to controls (Figures 6A,B) and respective analysis of cytotoxic CD8 T cells indicated a similar distribution but without statistical significance (Supplementary Table 5). Sex-specific differences regarding CD4- and CD8-naive and memory subsets were observed; that is, females in the obese and control groups had significantly more naive CD4 T cells vs. decreased T_CM_ and to a lesser extent also T_EM_ subsets than males before and after booster; for CD8 T cells, the respective differences between males and females were not significant (Supplementary Table 5).

#### T Regulatory Cells

T regulatory cells (CD4^+^/CD25^+^/FOXP3^+^) as percentages of CD4 T cells were present in similar frequencies in the obese and control groups before booster and significantly decreased after vaccination in both groups (Figure 6C).

#### T Follicular Helper Cells

**Figure 7:**
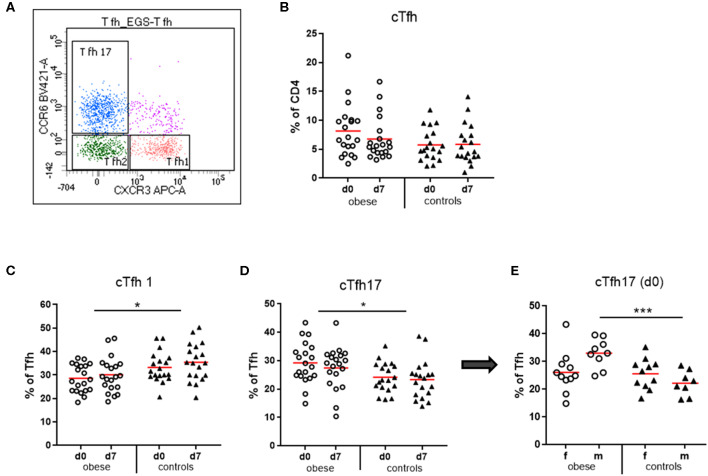
Quantification of circulating T follicular helper cells (cTfh) and cTfh1, cTfh2, and cTfh17 subsets. Peripheral blood mononuclear cells were stained with fluorochrome-labeled mAbs and analyzed on a BD FACS Canto II flow cytometer. **(A)** Scatterplot of total cTfh (CD3^+^/CD4^+^/CD45RA^−^/CXCR5^+^) with respective expression of CXCR3 and/or CCR6 to distinguish cTfh1, cTfh2, and cTfh17 subsets. CXCR3^+^CCR6^−^ cell are cTfh1 cells; CXCR3^−^CCR6^−^ are cTfh2 cells, and CXCR3^−^CCR6^+^ cells are cTfh17 cells. **(B)** Quantification of cTfh (CD3^+^/CD4^+^/CD45RA^−^/CXCR5+) before (d0) and 1 week (d7) after booster vaccination as % of total CD4^+^ T cells. **(C)** cTfh1 and **(D)** cTfh17 cells as % of total cTfh before (d0) and 1 week (d7) after booster; line is arithmetic mean. **(E)** cTfh17 cells as % of total cTfh before TBE booster (d0) in females and males. Analysis of variance with linear contrasts; *p ≤ 0.05; ***p ≤ 0.05.

Circulating T follicular helper cells (cTfh) as percent of CD4 T cells were quantified in our study population. We observed a trend toward increased cTfh in obese subjects before booster (*p* = 0.11), which declined after booster (*p* = 0.09), an observation not present in controls (Figure 7B). Analysis of the cTfh1, cTfh2, and cTfh17 cell subsets as percent of total cTfh (Figure 7) showed a significant increase of the cTfh17 subset at the expense of cTfh1 cells in the obese cohort. Quantification of cTfh17 cells according to sex revealed that in particular obese males had expanded cTfh17 populations before booster (Figures 7C–E).

### Total IgA

Total IgA levels were measured and found to be significantly elevated in the obese group (Supplementary Figure 3A).

### Reactogenicity

Our study participants reported local and systemic reactions to the TBE vaccination by use of a diary for 1 week after vaccine administration. Occurrence of pain, redness, itching, and swelling at the injection site were local reactions, and nausea, vomiting, headache, fatigue, joint pain, muscle pain, and fever were summarized under systemic side effects. Duration and intensity (one/mild, two/intermediate, three/strong) were documented and reporting of at least one of these local or systemic parameters accounted for occurrence of side effects. Overall, the incidence of local reactions to the TBE vaccination was similar in the obese and control groups, while there was a trend toward increased systemic reactions in obese (Table 3). Analysis according to sex showed that local reactions (mostly pain at the vaccination site) tended to be increased in obese males and female controls, although not statistically significant (Supplementary Figure 4A). The incidence of systemic reactions was comparable for men and women in both groups. Intensity and duration of local and systemic side effects were found to be similar in obese and controls (Supplementary Figures 5A,B).

**Table 3:** Incidence of local and systemic reactions.

**Reactogenicity**	**Obese**		**Controls**		***p***
	***n***	**%**	***n***	**%**	
**Local**					
Total	23/37	62.2	21/36	58.3	0.73
Male	12/17	70.6	7/16	43.8	0.12
Female	11/20	55.0	14/20	70.0	0.33
*p* m/f		*p =* 0.32		*p =* 0.10	
**Systemic**					
Total	20/37	54.1	13/36	36.1	0.12
Male	10/17	58.8	7/16	43.8	0.39
Female	10/20	50.0	6/20	30.0	0.20
*p* m/f		*p =* 0.59		*p =* 0.39	

Incidence of local and systemic reactions to intramuscularly applied TBE vaccine. Frequency of local and systemic reactions as n/group and as percentage for entire group (total), males, and females are indicated. Calculation of p values by general linear model with binomial counts and obesity and gender as factors; comparison by Wald χ^2^; *p ≤ 0.05.

## Discussion

Vaccine responsiveness in overweight and obese cohorts shows diverse outcomes compared to normal-weight populations. In order to clearly assess the effects of obesity on vaccine responses, we recruited subjects that differed strongly in terms of weight, that is, a severely obese cohort with average BMI 38.9 kg/m^2^ vs. lean controls with BMI 22.1 kg/m^2^. The mean age of subjects was younger than 50 years (mean, 45 years), and we therefore can exclude the influence of immune senescence on our results. The choice to test TBE vaccine was based on the fact that TBE virus is highly endemic in Austria, and sufficient protection of the growing obese population with potentially increased susceptibility is of clinical importance.

The results of the metabolic and hormone characterization of our cohorts confirm the described alterations in obese individuals, who presented with increased triglycerides, cholesterol ratios, and CRP levels. Leptin and insulin concentrations were also increased in the obese cohort, in particular in obese males (Figures 1A–F). Testosterone was significantly decreased in obese males (Figure 2). In normal-weight men, testosterone leads to—in comparison to women—lower leptin levels because dihydrotestosterone reduces the synthesis of transcripts encoding leptin (34). This is overruled in obese males—as seen in our study—because of the presences of high leptin levels that repress the steroidogenic gene expression in Leydig cells as shown by Landry et al. (35). This mutual influence of leptin and testosterone results in lower testosterone levels as observed in obese males of this study, and further reduction can occur because of aromatase-dependent conversion of testosterone to estrogen in obese WAT as described by Michalakis et al. (36). In our study, testosterone was elevated in obese females, which is in line with reports that insulin and leptin resistance lead to a dysregulation of the hypothalamic–pituitary–gonadal axis with increased testosterone synthesis in obese women (37). Because of these clearly evident metabolic and hormonal differences in our cohorts, this investigation was well-suited to detect differences in vaccine responsiveness due to an obese state.

Protection against vaccine-preventable diseases is important in obese individuals because of the described higher susceptibility to bacterial and viral infections. With respect to TBE virus, no data on the severity of infection or on the efficacy of TBE vaccination in obese subjects are available so far. The immunogenicity of vaccinations under obese conditions is particularly well-analyzed for annually applied influenza vaccine, where immediate and long-term responses have been investigated. Sheridan et al. (25) show that an initial higher-fold increase of influenza-specific Abs after 4 weeks is followed by a faster antibody decline until 12 months after booster, both correlating with BMI of the vaccinees. In accordance with this study, we observed very similar titer kinetics upon TBE booster vaccination. Prior to vaccination, NT titers were lower (though not significantly) in obese than in controls. However, the obese group showed a higher fold increase (d7 to d28) of Ab titers that was followed by a significantly faster decline until 6 months after booster (Figures 3A,B). Body mass index and also metabolic parameters, in particular insulin, correlated with the fold increase and decline rates of the TBE-specific Abs (Table 2A). An obese metabolic state with low-grade inflammation and high leptin and insulin levels obviously allows for a fast short-term Ab increase in a booster setting, but importantly is also associated with a faster Ab decline, which might limit the long-lasting protection. The strong correlation with insulin may qualify this parameter as a prediction marker for Ab kinetics under obese conditions.

The influence of sex on vaccine responses shows that females usually mount more robust humoral and cellular immune responses to vaccination and infection (38, 39). Sex hormones affect innate and adaptive immune responses due to estrogen receptors (ERα/β) being expressed on many immune cells. High estrogen levels in women lead to increased T_H_2 activation, expanded B-cell proliferation, and increased Ab production. Testosterone, in contrast, has inhibitory effects on generation of Abs (40). Accordingly, we saw these sex-specific differences in healthy controls in our recently published TBE booster study, where we compared vaccine responsiveness of allergic and healthy subjects (41). In the present study, we could show that this sex/gender aspect was reversed under obese conditions because in the obese group men showed a stronger fold increase of Abs than women 4 weeks after booster (*p* = 0.05, Figure 3B). Testosterone levels in males showed significant correlation with NT titers at baseline and correlated moderately with titer increase from d0 to d28 and Ab decline thereafter (Table 2B). As testosterone has been shown to directly act on immune cells by repressing transcription factors (e.g., FOS, JUN) involved in immune activation (40), the reduced testosterone in the obese males in our study was probably involved in the increased initial Ab rise and faster decline. Even though the decline rates at 6 months showed no sex-specific differences (Figure 3B), low testosterone might affect long-term decline, because NT titers prior to vaccination tended to be lower in obese vs. control males (GMT 210 vs. 299, not statistically significant). In addition to testosterone, also the metabolic parameters leptin and insulin might affect the antibody kinetic directly, as also obese women tended to have lower Abs before booster than normal-weight controls (GMT 244 vs. 319, not statistically significant). No direct effects of the estrogen levels on the antibody kinetic were seen in obese women.

The state of obesity also influenced distributions of lymphocyte subsets. Significantly increased CD19^+^ B cells in the obese cohort (Figure 5B) might be the result of permanently and strongly increased leptin levels. As B cells express the long form of LepR, that is, capable of leptin-signaling via the JAK-STAT pathway, leptin promotes B-cell homeostasis by inhibiting apoptosis and inducing cell-cycle entry through the activation of BCL2 and cyclin D1 expression. Leptin also influences B-cell development, because bone marrow of starved mice with low leptin levels contained less pro B, pre B, and immature B cells (6). In accordance, we observed increased naive B cells vs. decreased memory B cells in the obese group, which is in line with data from Frasca et al. (42), who also show more naive B cells vs. reduced B memory subsets in young and elderly obese subjects. B-cell subsets in obese responded differently to TBE booster vaccination: in healthy controls, naive B cells decreased at the expense of unswitched and switched B-memory cells, indicating the induction of TBE-specific memory B cells, whereas obese showed no such changes (Figures 5B1–B3). This, together with limited expansion of plasma blasts 1 week after vaccination (Figure 5C), might be causative for the poor maintenance of Ab titers in obese, because plasma blasts are precursors of Ab-secreting cells in peripheral blood and eventually of long-lived plasma cells (31).

T follicular helper cells are specialized in providing help to B cells for induction of long-lasting Ab responses. They are essential for germinal center formation in lymph nodes and recirculate in peripheral blood characterized as CD3^+^/CD4^+^/CD45RA^−^/CXCR5^+^ cells. These circulating resting memory Tfh cells comprise several populations with unique phenotypes and functions (43). Of the three described subsets (Figure 7A), Tfh2 and Tfh17 cells provide efficient help to naive and memory B cells, whereas Tfh1 cells lack this capacity. Moreover, Tfh subtype influences isotype switching: Tfh2 cells promote production of IgG and IgE, whereas Tfh17 cells induce IgG and in particular IgA secretion (44). In the by-trend increased total cTfh cells in the obese group, a significantly increased cTfh17 vs. a decreased cTfh1 subset was present (Figures 7C–E). Interestingly, patients with inflammatory autoimmune diseases with increased auto-Ab production (e.g., systemic lupus erythematosus) show similar frequency and distribution of Tfh cells (45). It is known that leptin propagates T_H_17 responses (6), and accordingly, we see expansion of cTfh17 cells in the obese group. This T_H_17 bias might be related to significantly increased serum IgA concentrations and rather elevated IL-17 production in SEB-stimulated PBMCs (Supplementary Figures 3A,B). The cTfh17subset was particularly prominent in obese males, where we observed a strong initial Ab increase.

The tendency toward reduced total CD3^+^ T cells in the obese cohort can be explained by significantly lower NK T cells and to a lesser extent lower CD8^+^ CTLs but not CD4^+^ T-helper cells (Supplementary Figures 2A–D). Invariant natural killer T (iNKT) cells are tissue resident and produce anti-inflammatory/regulatory cytokines to maintain adipose tissue homeostasis. Depletion of iNKT cells in obese WAT contributes to the inflammatory environment (46), and our finding of fewer circulating NKT cells could indicate that loss of iNKT in obese WAT is also reflected in the peripheral blood. We found CD8^+^ T cells non-significantly decreased in obese, which is line with reports that CD8^+^ T cells accumulate in WAT under obese conditions and rather expand locally without significant peripheral changes (47). The numbers of CD4^+^ T-helper cells were similar in both obese and normal-weight vaccination groups, but their phenotype and function appear altered in the obese cohort. Higher TBE-specific and mitogen-induced production of IL-2 (Figures 4A,C) might reflect enhanced leptin-dependent naive T-cell proliferation (48), whereas by-trend increased TBE-specific IFN-γ levels (Figure 4B) indicate a proinflammatory T_H_1 phenotype due to influence of leptin (6, 47).

The T effector memory subsets of CD4 and non-significantly also of CD8 T cells were decreased in the obese subjects, whereas central memory compartments were rather increased (Figures 6A,B and Supplementary Table 4). Because effector memory T cells home to peripheral tissues, obese individuals might have fewer tissue-resident T_EM_ cells specific for various previously encountered antigens, which could relate to a generally increased susceptibility for infections in obese individuals. Of note, TBE booster vaccination had no influence on the distributions of CD4/CD8 T effector and central memory populations.

Leptin signaling in Tregs (CD4^+^CD25^+^/FOXP3^+^) leads to reduced Treg proliferation (48), and lower numbers of Tregs are present in WAT, but also in peripheral blood of obese individuals (49). In our study population, Tregs as percent of CD4 T cells did not differ between groups, but we observed decreased percentages of Tregs after booster in both groups (Figure 6C), which is in accordance with our previous findings of reduced Tregs during mounting of vaccine responses (27, 41).

Regarding vaccine reactogenicity during obesity, we observed that the incidence of systemic reactions to TBE booster vaccination was slightly higher in the obese cohort (Table 3). Because common inflammatory markers increase after administration of adjuvanted vaccines (50), the *a priori* proinflammatory milieu associated with obesity might lead to more systemic vaccine side effects. With regard to local side effects, it has been described that females are often more affected after vaccination (51, 52), which was also seen in the healthy controls of our current study. In the obese cohort, however, men reported more local pain, which seems in line with the initially higher vaccine responses, as it has been described that strong vaccine responsiveness is often accompanied by increased reactogenicity (52). Moreover, the route of application might also influence reactogenicity, as we previously described that subcutaneous TBE vaccination is associated with more side effects—even if immunogenicity is unaffected by the route of application (53). Thus, possible unintentional subcutaneous administration in obese individuals, for example, due to insufficient needle length, might also account for increased local reactions.

**Figure 8:**
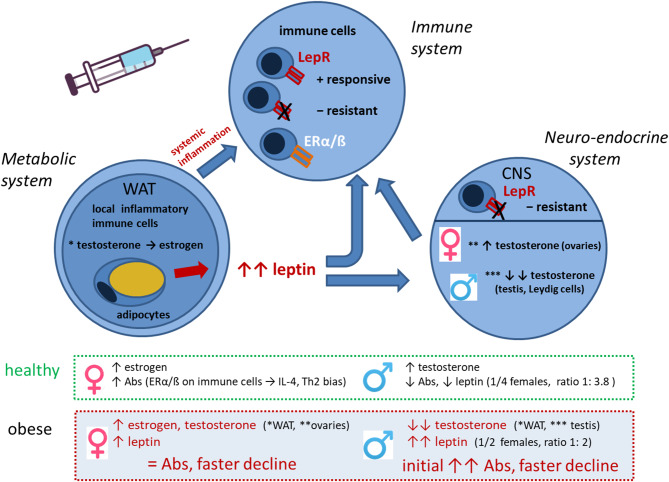
Interplay of metabolic, neuro-endocrine and immune system in the obese state in the context of vaccination. Interactions between metabolic, neuro-endocrine and immune system in the obese state that influence vaccine responses in a sex-dependent manner. Abbreviations: WAT, white adipose tissue; LepR, leptin receptor; ERα/β, estrogen receptor α/β; CNS, central nervous system; Abs, antibodies; Th2, T helper cells type 2.

Taken together, the data obtained in this study indicate a complex interplay of the metabolic, endocrine, and immune system under obese conditions, which influences responsiveness to vaccination (Figure 8). Obesity is a state of chronic low-grade inflammation in adipose tissues due to loss of metabolic homeostasis with systemic effects on various organs and also the immune system. The high leptin levels in obesity have immune-stimulatory effects on many immune cells, but due to leptin resistance and altered immune cell functionality, also dysregulated/suppressive immune responses occur. Obesity-associated alterations in male hormones further influence the axis between the metabolic and immune system. We show that an obese state with low-grade inflammation and high leptin/insulin levels allows for increased short-term Ab response but is followed by a faster Ab decline. Among the sex hormones, testosterone seems to have a particularly strong influence on Ab kinetics and reactogenicity in obese conditions.

Because faster decline rates could lead to reduced long-term protection, we now investigate in a second part of the trial whether NT titers after 3 years are below protective levels, that is, before the standard booster interval of 5 years. Our study cannot predict the immune responsiveness to TBE vaccination in TBE-naive obese individuals. Based on other studies with hepatitis A and B or tetanus vaccine that emphasize impaired vaccine responses to neoantigens, we clearly see a need for investigation of primary TBE vaccination in obese subjects.

## Data Availability Statement

The datasets generated for this study are available on request to the corresponding author.

## Ethics Statement

The studies involving human participants were reviewed and approved by Ethikkommission der Medizinischen Universität Wien Borschkegasse 8b/E06 A-1090 Wien. The patients/participants provided their written informed consent to participate in this study.

## Author Contributions

UW conceived the clinical trial. CS-F and IZ facilitated the recruitment of the study subjects and IZ obtained ethics approval. E-MP, together with AW and CS-F administered the study. AG contributed to analyzing data and performed preliminary statistical analyses. EG-S performed experiments, analyzed data and did preliminary statistical analyses. KS provided TBE NT results and CB measurement of total immunoglobulins. MK contributed statistical planning and did the final statistical analyses. EG-S and UW wrote the manuscript.

## Conflict of Interest

E-MP is a consultant for Pfizer (TBE) and Valneva; KS's institution, the Department of Virology, received research support from Pfizer for TBE epidemiology studies in Austria; MK was a board member for Baxter and provided consultancy for Baxter, Pfizer and Sanofi; UW was CSO of Imugene, Ltd. until Sept. 2018 and received funding to the Institute from GSK, Pfizer and Themis. The remaining authors declare that the research was conducted in the absence of any commercial or financial relationships that could be construed as a potential conflict of interest.
