# Supplementary material for: Obesity and Sex Affect the Immune Responses to Tick-Borne Encephalitis Booster Vaccination
Source: Front Immunol. 2020 May 27;11:860. doi: 10.3389/fimmu.2020.00860 (PMC7266951; doi:10.3389/fimmu.2020.00860)

Supp. Table 1: *General study eligibility*

Inclusion Criteria:

- participants of both sexes between 18 and 60 years of age
- completed primary TBE immunization and at least 1 booster vaccination
- willingness to sign written informed consent form

Exclusion Criteria:

- age < 18 and > 60 years
- BMI 25-30
- previous TBE infection
- pregnancy or breast feeding
- acute infection on day of inclusion (day 0), body temperature > 37,9°C
- concomitant medications: systemic cortisone therapy, chemotherapy, immunotherapy (allergy), immunosuppressive therapy 4 weeks prior or during the study
- administration of other vaccines 4 weeks before/after day 0
- planned surgery within 2 weeks before/after TBE booster
- any contraindication to administration of FSME-Immun® vaccine according to manufacturer's instructions
- malignant diseases within 5 years prior to the study
- autoimmune diseases
- kidney insufficiency, dialysis
- drug addiction
- plasma donor
- receipt of blood transfusions or immunoglobulins within 3 months prior to study entry
- severe disease with hospitalization or surgery 3 months before or during the study
- participation in a clinical trial simultaneously to visit 1-4 with receipt of vaccination and/or investigational product within one month before booster

Supp. Table 2: *Changes in metabolic parameters 4 weeks after TBE booster*

Concentrations of triglycerides, total cholesterol, HDL cholesterol, glucose (all mg/dl); insulin (mIU/L), fructosamine (µmol/L) and hsCRP (mg/dl) were measured before (d0) and 4 weeks (d28) after booster vaccination. Cholesterol ratio was calculated as total cholesterol/HDL cholesterol and LDL cholesterol was calculated according to the Friedewald formula; values are indicated as geometric mean (GM) concentrations with 95% CI.

Abbreviations: tgl, triglycerides; chol, total cholesterol; LDL, LDL cholesterol; HDL, HDL cholesterol; chol ratio, cholesterol ratio; fruct, fructosamine; L 95% CI, lower 95% confidence interval; U 95% CI, upper 95% confidence interval.

			tgl	chol	LDL	HDL	chol ratio	glucose	insulin	fruct	hsCRP
**female**	**obese**	**d0**	116,9	197,6	119,3	53,9	3,67	94,7	16,38	232,1	0,43
		L 95% CI	94,8	181,1	102,9	47,6	3,2	87,8	12,57	221,8	0,27
		U 95% CI	144,2	215,5	138,3	61,0	4,21	102,1	21,35	242,9	0,66
	**obese**	**d28**	106,6	188,5	111,0	52,9	3,56	95,5	13,84	225,7	0,31
		L 95% CI	86,0	174,7	97,8	46,8	3,11	88,1	10,58	217,0	0,24
		U 95% CI	132,1	203,4	126,0	59,8	4,08	103,5	18,12	234,8	0,40
	**controls**	**d0**	77,3	225,0	118,5	85,7	2,61	86,6	7,66	258,7	0,04
		L 95% CI	62,0	205,5	101,5	75,3	2,26	80,3	5,88	246,9	0,02
		U 95% CI	96,3	246,4	138,4	97,6	3,02	93,4	9,98	271,0	0,06
	**controls**	**d28**	72,8	218,3	114,2	84,6	2,58	85,0	6,20	243,2	0,16
		L 95% CI	58,2	201,6	100,0	74,4	2,24	78,5	4,74	233,6	0,13
		U 95% CI	91,2	236,4	130,4	96,1	2,98	92,1	8,11	253,3	0,21
**male**	**obese**	**d0**	152,9	196,8	137,6	40,9	4,82	97,0	22,84	229,4	0,32
		L 95% CI	121,8	179,2	117,2	35,7	4,15	89,3	17,15	218,4	0,20
		U 95% CI	191,8	216,2	161,4	46,7	5,6	105,2	30,42	241,0	0,52
	**obese**	**d28**	164,0	190,3	126,8	40,1	4,76	104,8	23,96	230,9	0,27
		L 95% CI	130,0	175,3	110,5	35,1	4,11	96,1	17,91	221,2	0,21
		U 95% CI	206,8	206,6	145,4	45,8	5,5	114,4	32,06	241,0	0,35
	**controls**	**d0**	88,9	204,6	117,9	62,0	3,31	89,4	6,53	247,2	0,02
		L 95% CI	69,8	185,2	99,4	53,8	2,82	82,1	4,85	234,9	0,01
		U 95% CI	113,2	226,1	139,7	71,5	3,88	97,3	8,79	260,1	0,04
	**controls**	**d28**	96,1	208,6	131,9	56,2	3,72	87,2	6,70	236,2	0,15
		L 95% CI	75,0	191,2	114,0	48,8	3,19	79,7	4,95	226,0	0,11
		U 95% CI	123,0	227,7	152,7	64,7	4,35	95,5	9,06	246,9	0,20

Supp. Table 3: *Serum hormone levels*

Concentrations of the sex hormones testosterone, estrogen and progesterone (in ng/ml) and luteinizing hormone (LH) and follicle-stimulating hormone (FSH) (in mU/ml) indicated as geometric mean (GM) concentrations with 95% CI.

Abbreviations: L 95% CI, lower 95% confidence interval; U 95% CI, upper 95% confidence interval.

	controls male	L 95% CI	U 95% CI	controls female	L 95% CI	U 95% CI
**testosterone**	6,05	5,16	7,10	0,13	0,09	0,19
**estrogen**	28,92	24,76	33,78	19,24	9,92	37,33
**progesterone**	0,18	0,12	0,28	0,31	0,14	0,73
**FSH**	5,52	4,38	6,95	17,89	7,45	42,95
**LH**	5,29	4,47	6,25	9,91	3,89	25,24
	**obese male**			**obese female**		
**testosterone**	3,42	2,87	4,07	0,22	0,17	0,29
**estrogen**	29,61	25,98	33,75	29,92	18,11	49,41
**progesterone**	0,29	0,20	0,44	0,30	0,15	0,60
**FSH**	4,43	3,57	5,51	16,63	9,80	28,22
**LH**	5,16	4,23	6,28	12,46	7,82	19,87

Supp. Table 4: *Differences in total B cells, naïve and memory B subsets and plasma blasts according to sex*

PBMCs were stained with fluorochrome-labeled mAbs and analyzed on a BD FACS Canto II flow cytometer. Distributions of total CD19+ B cells and plasma blasts as % of total lymphocytes as well as of naïve, un-switched memory, switched memory and double-negative (DN) B subsets as % of total B cells before (d0) and one week (d7) after TBE booster vaccination are listed. Values for the entire group (all) and separately for females (F) and males (M) before (d0) and 1 week (d7) after booster are indicated.

Abbreviations: ly, lymphocytes; tot, total; unsw, unswitched; sw, switched; DN, double negative; PB, plasma blasts

		total B as % of ly		naive as % tot B		unsw as % tot B		sw as % tot B		DN as % tot B		PB as % of ly	
		d0	d7	d0	d7	d0	d7	d0	d7	d0	d7	d0	d7
obese	all	10,58	10,56	67,13	68,01	15,16	14,18	13,86	14,11	3,84	3,71	1,69	1,89
	F	10,25	10,40	69,03	69,30	13,85	13,93	13,36	13,32	3,76	3,46	1,69	1,88
	M	10,97	10,74	64,88	66,47	16,72	14,47	14,46	15,05	3,94	4,01	1,69	1,91
controls	all	8,70	7,83	60,97	59,07	18,41	19,57	15,60	16,70	5,01	4,65	1,61	2,47
	F	8,94	7,80	62,66	60,07	15,78	17,73	15,76	17,05	5,80	5,16	1,40	2,49
	M	8,37	7,87	58,68	57,73	21,98	22,07	15,40	16,24	3,94	3,97	1,90	2,45

Supp. Table 5: *Differences in CD4 and CD8 T cell naïve and memory subsets according to sex*

PBMCs were stained with fluorochrome-labeled mAbs and analyzed on a BD FACS Canto II flow cytometer. Distributions of CD3+/CD4+ helper T cells and CD3+/CD8+ cytotoxic T cells as % of total lymphocytes. T naïve, T central memory (T_{CM}), T effector memory (T_{EM}) und T effector memory RA (T_{EMRA}) subsets as % of total CD4 and total CD8 T cells respectively are listed. Values for the entire group (all) and separately for females (F) and males (M) before (d0) and 1 week (d7) after booster are indicated.

Abbreviations: ly, lymphocytes; p sex, p value in respect to sex differences.

ANOVA with linear contrasts; *p≤0.05, **p≤0.01

		CD4 as % of ly		CD4 naive as % CD4		CD4 T_{CM} as % CD4		CD4 T_{EM} as % CD4		CD4 T_{EMRA} as % CD4	
		**d0**	**d7**	**d0**	**d7**	**d0**	**d7**	**d0**	**d7**	**d0**	**d7**
**obese**	**all**	45,4	43,7	51,7	51,5	34,5	33,7	10,6	11,2	3,2	3,6
	**F**	48,7	46,0	54,6	54,2	30,9	30,5	10,5	10,9	4,0	4,4
	**M**	41,3	40,8	48,0	48,1	39,0	37,8	10,7	11,6	2,3	2,6
**controls**	**all**	44,5	43,8	52,5	52,2	28,9	29,0	15,1	15,2	3,4	3,6
	**F**	46,4	46,1	55,1	55,1	27,0	27,4	14,7	14,0	3,3	3,5
	**M**	41,8	40,8	49,1	48,2	31,6	31,3	15,7	16,9	3,6	3,7
	**p sex**	**p=0.005	*p=0.01	*p=0.02	*p=0.01	*p=0.03	p=0.05	p=0.12	*p=0.04		
		**CD8 as % of ly**		**CD8 naive as % CD8**		**CD8 T_{CM} as % CD8**		**CD8 T_{EM} as % CD8**		**CD8 T_{EMRA} as % CD8**	
		**d0**	**d7**	**d0**	**d7**	**d0**	**d7**	**d0**	**d7**	**d0**	**d7**
**obese**	**all**	17,2	17,3	38,0	37,1	14,3	13,3	24,3	23,6	23,5	26,1
	**F**	16,3	15,9	41,3	40,2	12,8	12,4	21,7	21,6	24,3	25,9
	**M**	18,3	19,0	33,8	33,3	16,1	14,4	27,6	26,0	22,4	26,3
**controls**	**all**	19,4	19,8	39,5	38,3	10,2	10,5	27,7	27,9	22,6	23,3
	**F**	19,0	19,7	41,5	40,2	9,2	10,4	27,1	26,0	22,2	23,4
	**M**	19,9	19,9	36,7	35,7	11,5	10,7	28,5	30,5	23,3	23,1
	**p sex**	p=0.59	p=0.45	p=0.14	p=0.17	p=0.18	p=0.64	p=0.17	p=0.10		

Supp. Fig. 1: *Study design*

Flow-diagram of study design and study participants from screening to evaluation.

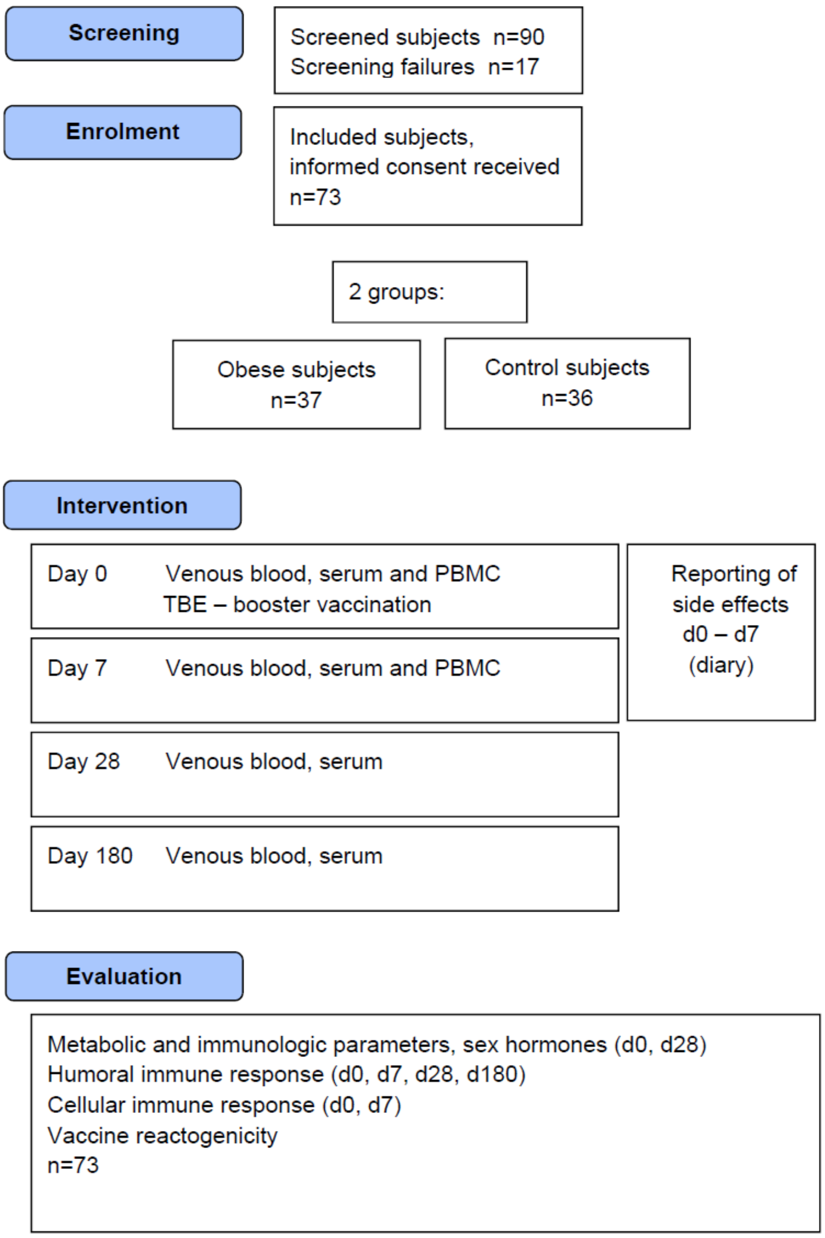

Supp. Fig. 2: *Quantification of T lymphocytes and T cell subsets*

PBMC were stained with fluorochrome-labeled mAbs and analyzed on a BD FACS Canto II flow cytometer. (A) CD3+ total T-cells (B) CD3+/CD4+ T-helper cells and (C) CD3+/CD8+ cytotoxic T-cells, and D) calculated CD3+/CD4-/CD8- NK T cells before (d0) and 1 week (d7) after booster vaccination as % of lymphocytes in the two investigated groups; line is arithmetic mean.

ANOVA with linear contrasts; * p≤0.05

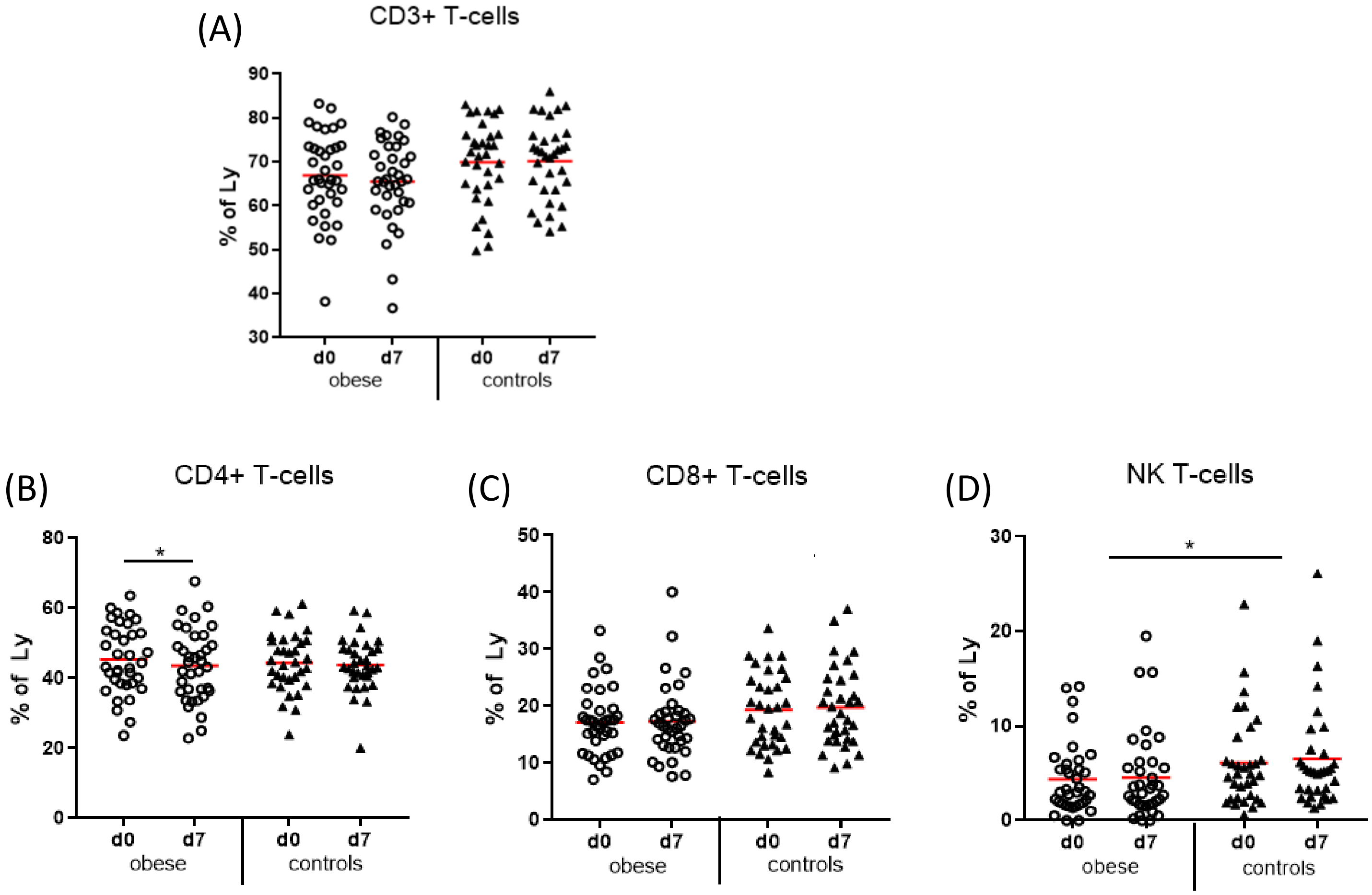

Supp. Fig. 3: *Total IgA & SEB-specific IL-17 production*

A) Concentrations of total serum immunoglobulin A (IgA) in obese and control cohort measured before (d0) and 4 weeks after vaccination (d28); in mg/dl as geometric mean (GM) with 95% CI.

ANOVA with linear contrasts; ** $p \leq 0.01$

B) IL-17 concentrations (pg/ml) were measured in PBMC culture supernatants with Luminex technology. PBMC obtained before (d0) and 1 week (d7) after booster were incubated with 1 µg/ml *Staphylococcus* Enterotoxin B for 48 h. Geometric mean (GM) concentrations (pg/ml) with 95% CI.

ANOVA with linear contrasts;

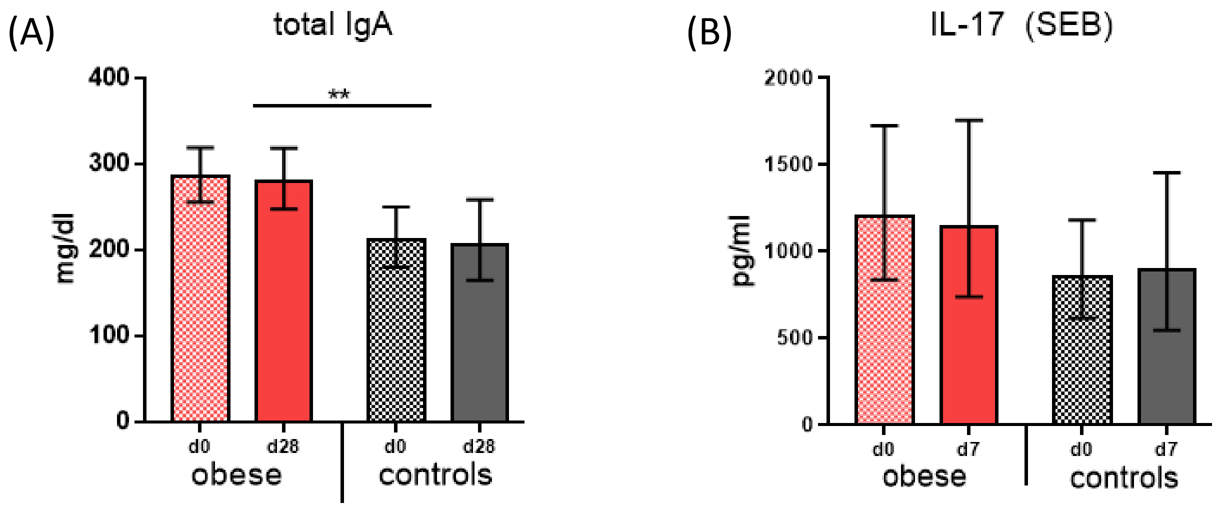

Obesity, sex and vaccine responsiveness

Supp. Fig. 4: *Incidence of single local and systemic side effects*

Incidence of (A) single local and (B) single systemic side effects according to sex

(A)

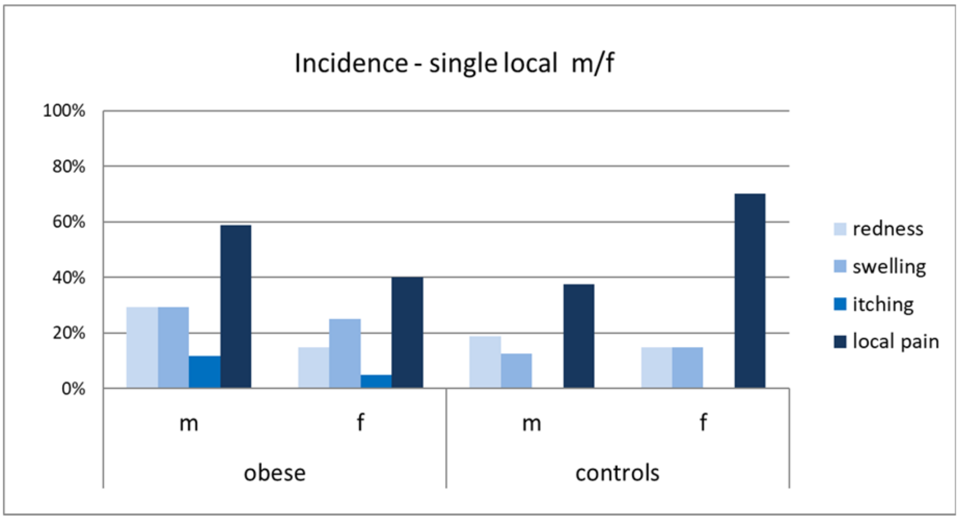

(B)

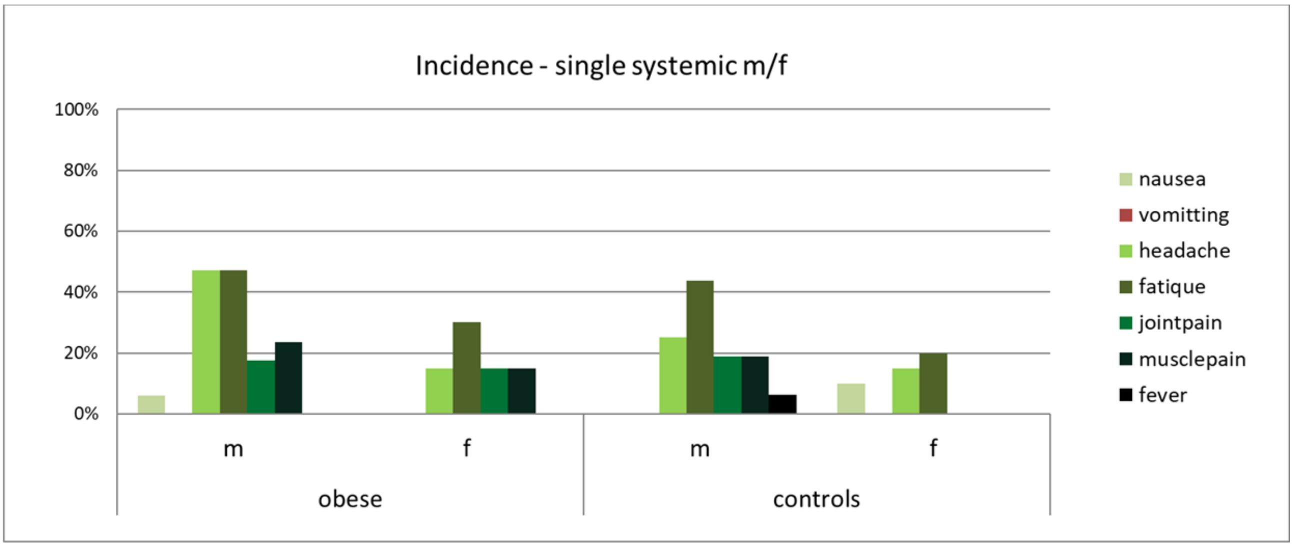

Supp. Fig. 5: *Intensity and duration of local and systemic side effects*

(A) Intensity and duration of local side effects according to sex

Arithmetic means of intensities of local side effects after vaccination for males and females over a 7-day period; error bar is SEM. Local effects are pain, redness, itching and swelling at the injection site. Intensity classified as one/mild, two/intermediate, three/strong.

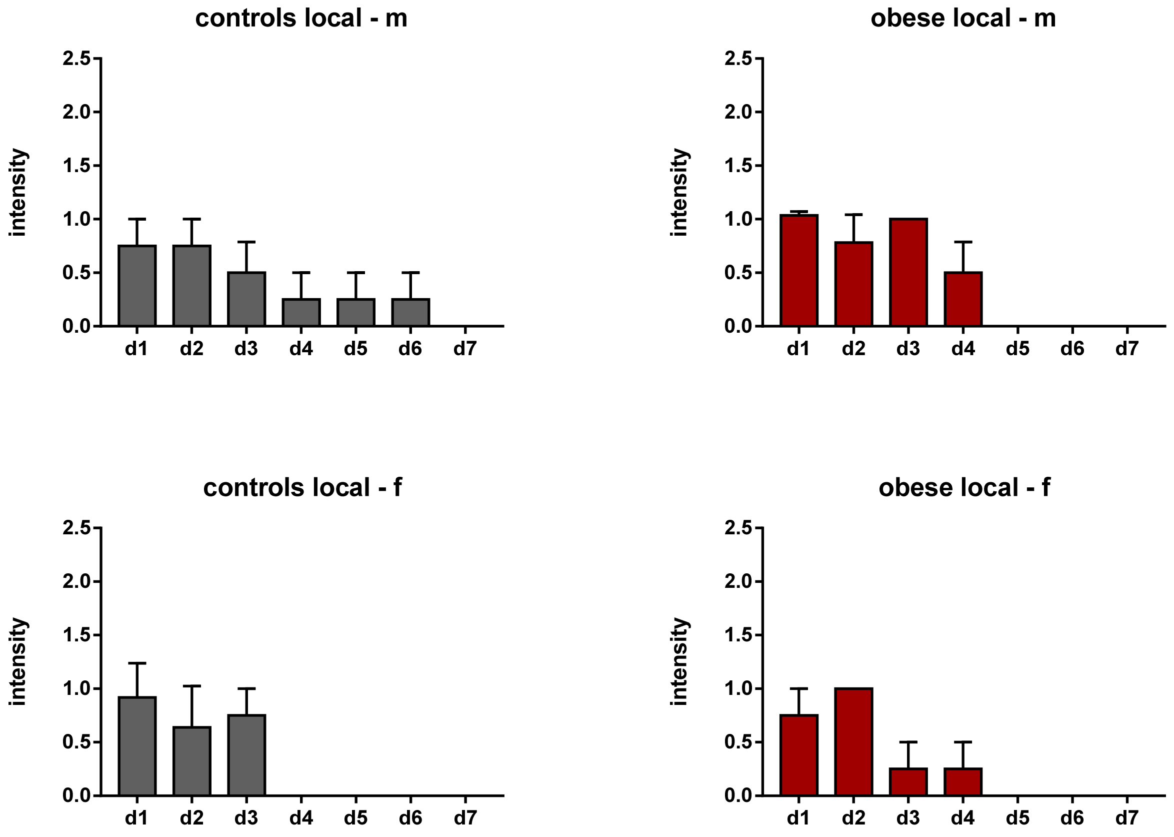

(B) Intensity and duration of systemic side effects according to sex

Arithmetic means of intensities of systemic side effects after vaccination for males and females over a 7-day period; error bar is SEM. Systemic effects are nausea, vomiting, headache, fatigue, joint pain, muscle pain and fever. Intensity classified as one/mild, two/intermediate, three/strong.

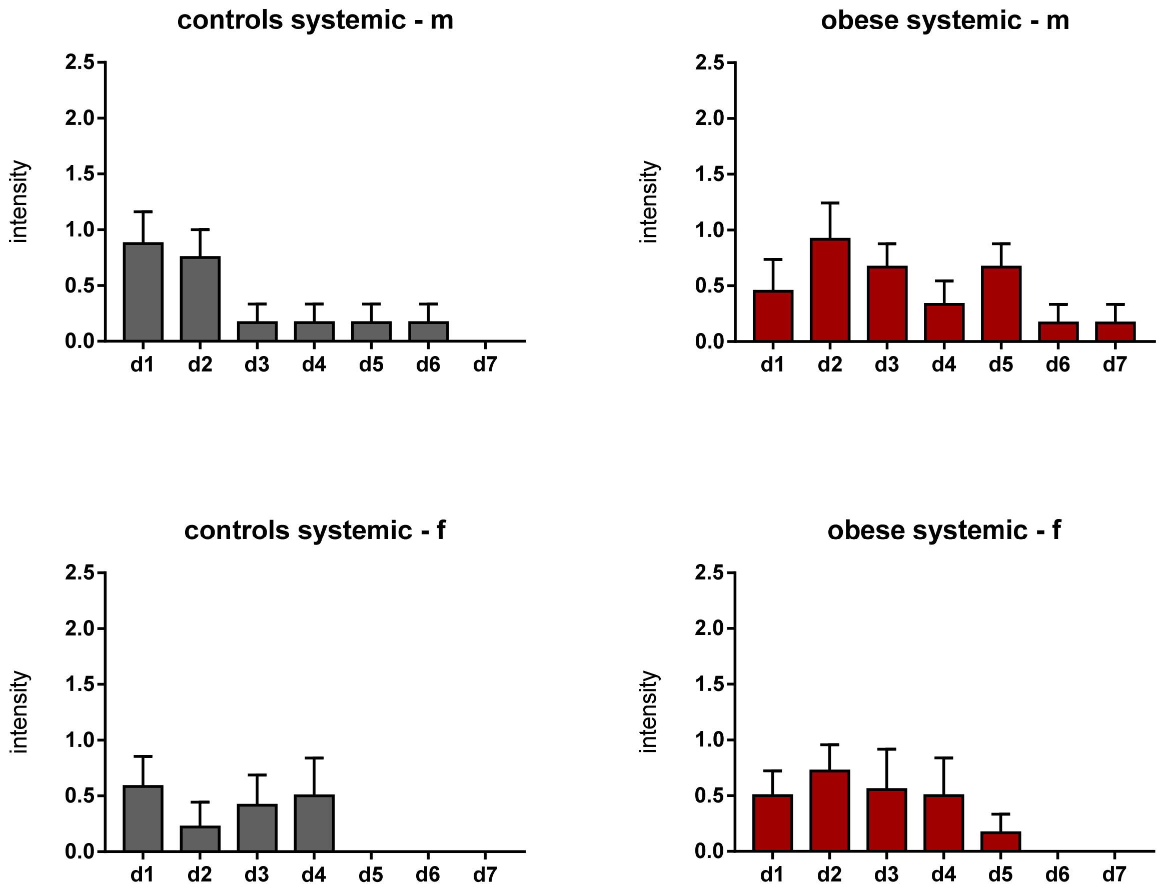

Supplement: Supplementary file 1 [file Data_Sheet_1.pdf]
